# Artificial intelligence in prehospital emergency care systems in low- and middle-income countries: cure or curiosity? Insights from a qualitative study

**DOI:** 10.3389/fpubh.2025.1632029

**Published:** 2025-10-01

**Authors:** Odhran Mallon, Freddy Lippert, Eva Pilot

**Affiliations:** ^1^Faculty of Health, Medicine and Life Sciences, Maastricht University, Maastricht, Netherlands; ^2^Falck, Copenhagen, Denmark; ^3^Faculty of Medical Sciences, Newcastle University, Newcastle upon Tyne, United Kingdom; ^4^Faculty of Health and Medical Sciences, University of Copenhagen, Copenhagen, Denmark

**Keywords:** artificial intelligence, machine learning, prehospital, emergency medical services, LMIC, emergency patient care

## Abstract

**Introduction:**

The adoption of artificial intelligence (AI) in prehospital emergency medicine has predominantly been confined to high-income countries, leaving untapped potential in low- and middle-income countries (LMICs). AI holds promise to address challenges in out-of-hospital care within LMICs, thereby narrowing global health inequities. To achieve this, it is important to understand the success factors and challenges in implementing AI models in these settings.

**Methods:**

A scoping review of peer-reviewed studies and semi-structured expert interviews were conducted to identify key insights into AI deployment in LMIC prehospital care. Data collection occurred between June and October 2024. Using thematic analysis, qualitative data was systematically coded to extract common themes within the studies and interview transcripts. Themes were then summarised narratively and supplemented with illustrative quotations in table format.

**Results:**

From 16 articles and nine expert interview transcripts, five core themes emerged: (1) the rapid, iterative development of AI technologies; (2) the necessity of high-quality, representative, and unbiased data; (3) resource gaps impacting AI implementation; (4) the imperative of integrating human-centred design principles; and (5) the importance of cultural and contextual relevance for AI acceptance.

**Conclusion:**

Additional focus on these areas can help drive the sustainable utilisation and ensuing development of AI in these environments. Strengthening collaboration and education amongst stakeholders and focusing on local needs and user engagement will be critical to promoting future success. Moving forwards, research should emphasise the importance of evidence-based AI development and appropriate data utilisation to ensure equitable, impactful solutions for all users.

## 1 Introduction

Artificial intelligence (AI) can be defined as technology’s ability to learn and perform tasks that simulate human behaviours, often at faster speeds and with higher accuracy than humans (1, 2). The recent exponential growth of this technology and a global appreciation of the efficiency gains it can generate, coincide with a time where there is an urgent global need for improvements in healthcare systems. Unfortunately, most current research investigating the use of AI in healthcare services is conducted in and for the benefit of high-income countries (HICs) (3), widening the health equity gap between these countries and low- and middle-income countries (LMICs). However, AI has the potential to transform the pursuit of global health equity by expanding essential healthcare coverage to underserved populations, including those in LMICs (4). Although there is comparatively little published research on AI within the healthcare settings of LMICs, this does not reflect current efforts in this field (1). Advances in mobile phone accessibility in rural and urban areas, investments in electronic health records (EHR) and the continued development of Information Technology (IT) infrastructure mean these countries have the capacity to utilise AI within their healthcare systems (1, 5, 6).

One area of healthcare that can particularly benefit from AI is emergency care. This is defined as “the care administered to a patient within the first few minutes or hours of suffering an acute and potentially life-threatening disease or injury” (7). The overall intention of providing emergency care is to minimise complications, including premature mortality and permanent disability (8). Emergency care has the greatest chance of achieving this goal when it is administered within the first hour of the injury or illness, during a period of time known as the golden hour (9). The golden hour usually occurs outside a hospital or healthcare facility, hence the ability of the prehospital emergency medical services (EMS) to react positively to unexpected casualties can have a significant impact on patient morbidity and mortality (9, 10). Moreover, the initial links in the “chain of survival” such as early recognition and call for help, contribute significantly more to improving patient outcomes than the later, advanced care links (11). Some researchers believe that AI can help overcome some of the barriers commonly associated with prehospital care in low-resource settings, such as shortages in skilled healthcare workers, a lack of medical equipment for diagnosis and management, and an absence of dedicated emergency vehicles. Addressing these challenges can reduce the gap in global health inequities (12–14), with AI models allowing EMS to more efficiently assess patients and promote the democratisation of clinical expertise (15, 16). This can help reduce the burden on EMS personnel and healthcare services while simultaneously improving patient outcomes (15, 17).

Within prehospital emergency care systems (PECS) in LMICs however, there is a lack of literature assessing the current broad applications of AI and the types of AI commonly utilised in these settings. Understanding AI’s current use in LMICs is important to ensure future systems are correctly integrated into the local context. Interventions designed for HICs cannot necessarily be transplanted into the PECS of LMICs. Local context plays a central role in the implementation of effective and sustainable health interventions (4, 18), meaning global health solutions cannot and should not be simply transferred into different communities (19, 20). Similarly, EMS models of care designed for use in HICs often are not sustainable solutions for LMICs (21).

This study seeks to address this research gap by firstly, identifying themes that exist in the current literature base of AI use within the PECS of LMICs and secondly, by consulting experts in this domain through qualitative interviews to provide an overview in AI utilisation in this field. This topic aligns with research recommendations by Masoumian Hosseini et al. (22), who advised increased focus on the different types of AI systems used and their interactions with healthcare professionals (and patients) to reconnect the gap between AI research and practice. By highlighting the outcomes and lessons learnt from previous interventions and their researchers, as well as exploring important considerations and upcoming challenges, future AI developers and prehospital personnel can ensure new models benefit local prehospital emergency patients, systems and staff while remaining contextually relevant in the long-term future (23).

## 2 Methods

This qualitative scoping review study has been reported according to the Consolidated Criteria for Reporting Qualitative Research (COREQ) guidelines (see Supplementary material for COREQ Checklist) (24). The qualitative scoping review study consists of thematic analysis of two distinct datasets: literature identified through scoping review, and transcripts of the consultations with field experts (25). This study will seek to answer the overarching research question: what is the current state of artificial intelligence utilisation in prehospital emergency care systems in LMICs, and what are the implications for future development? The qualitative scoping review protocol for this study was developed and registered with the Open Science Framework in June 2024 (26). Data collection of the literature and expert interviews was done independently, but datasets were combined for thematic analysis. The literature collected via scoping review for thematic analysis in this study, and the methodology used, is comprehensively described in the study by Mallon et al. (27). The literature collection process for the scoping review can also be seen in the PRISMA flowchart in the Supplementary material. In this study, all but one of the interviews were conducted by OM, who had not met any of the participants before commencing this study. As a male medical student studying for a Master’s degree in a healthcare-related topic (at the time of the study) from a HIC who has limited previous experience working in emergency and prehospital care in sub-Saharan Africa, the main researcher OM has made a conscious effort to ensure his previous experiences and personal perspectives did not infiltrate the data analysis process. OM has an interest in prehospital and emergency medicine and was undertaking this work as part of a Master’s project. FL and EP are experienced researchers from HICs with previous involvement in studies focusing on emergency medicine. As mentioned above, one interview was conducted by another researcher in a different language using the same interview question guide as the other interviews, and a transcript was translated to English by this interviewer.

Participants were selected and invited for interview via email through a combination of purposive and snowball sampling. This ensured a range of relevant perspectives from participants who possess an understanding of the topic, allowing research aims to be comprehensively addressed (28, 29). Participants were selected based on their experience working with or alongside AI tools designed for use in PECS, with nearly all participants having some relevant experience in LMICs. The sample size of nine was determined based off recommendations provided by Terry et al. of between six and 15 interviews for a Master’s project (30). The limited number of participants in this study reflects that this field is still an emerging research area, with limited experts having appropriate experience to act as participants in this study. The existing researchers in this field are also still establishing research networks and therefore may have been missed by our sampling methods. Several potential participants contacted via snowball sampling declined to participate as they did not feel they had enough experience in the field. Interviews were conducted online or at the workplace of the participant, depending on their preference and convenience. This ensured a professional but comfortable environment for participants.

Data collection took place between June and August 2024 and was analysed between July and October 2024. Participants took part in a one-off semi-structured interview lasting approximately 40 min. Only the interviewer and the participant were present during the interviews. Informed consent for interview and audio recording was acquired before the interview began. The researcher was equipped with a brief interview guide, a notebook for general notetaking during the interview, and an audio recording device. Interview guides were composed to include open questions structured around the overarching research question. This guide was reviewed and approved by all authors before beginning the interview process and further refined throughout the interviews. A copy of the final interview guide can be found in the Supplementary material. Experts were asked to reflect on their experiences in this field, and provide their opinions on the strengths, limitations and potential pitfalls in developing future AI models to address PECS in LMICs. Data collected from all expert consultations was qualitatively analysed from an interpretivist point of view using the thematic analysis method outlined by Braun and Clarke (31). Constructionist positions, which can be characterised as a form of interpretivism (32), were applied during the thematic analysis method. These positions make it difficult to ascertain data saturation based solely off sample size (33), and therefore data saturation is not reported in this study. Furthermore, this study primarily seeks to provide an overview of the topic by combining findings from literature and experts. Due to this adapted method, the sample size is appropriate for the intentions of the study and data saturation is less important than would be required for a standalone qualitative analysis of interviews.

Data was transcribed verbatim from audio recordings of the interview and checked by the interviewer to ensure integrity. Transcripts were not returned to the participants. Identifiable information including names and locations were removed, and each expert was assigned an ID number. These ID numbers are used throughout the results section to refer to the individual expert interviews. The anonymised transcripts and the articles identified in the scoping review were uploaded to ATLAS.ti 24 Mac (Version 24.1) for thematic analysis by OM (34). The data was then analysed using the iterative process of thematic analysis discussed by Braun and Clarke (31) to produce a narrative synthesis (34). This involved initial familiarisation with the data before generating the initial codes. Coding was conducted by one coder and based on interesting data features that can be meaningfully interpreted within the transcripts and articles (31). These codes were integrated into themes derived from the selected articles and alongside the transcripts of expert consultations. These identified themes were then discussed between all the authors to ensure all authors were in agreement, before being further refined (31). To ensure trustworthiness, supporting quotations of important themes identified during thematic analysis are included in the final manuscript (24), however participants did not provide feedback on the findings. Qualitative themes identified through thematic analysis of the literature review and expert consultations are discussed as part of the narrative summary with selected quotations displayed in table format.

## 3 Results

This study involved thematic analysis of the available literature identified by scoping review, and the consultation of nine experts. Table 1 provides a brief, anonymised overview of each of the nine expert’s individual characteristics. There were 16 studies identified through scoping review that were included for thematic analysis in this study (27). The majority of studies were from China (35–43), with the other seven countries with studies having only one study. In addition, all but one of the included studies were retrospective (42), with most studies conducted as cohort studies and three using simulation modelling (38, 44, 45). Further characteristics of the selected studies that were analysed can be seen in the previous study by Mallon et al. (27).

**Table 1:** Individual characteristics of experts that were consulted.

ID	Interview date	Continent	Relevant experience
1	June 2024	South America	Head of AI at an international prehospital healthcare company with experience working in an LMIC.
2	June 2024	Europe	Former associate professor and current partner at a technology solutions company that specialises in emergency and healthcare services globally.
3	June 2024	Europe	Medical doctor with previous experience using machine learning (ML) to predict emergency conditions in prehospital settings.
4	June 2024	Europe	Senior researcher in emergency medicine with extensive research experience using artificial intelligence in emergency dispatch calls.
5	July 2024	Asia	Director and professor of prehospital emergency medicine with extensive research in AI. Chairperson of a study collecting data on an emergency condition.
6	July 2024	Africa	CEO and managing director of a company providing telematics and networking ambulances in sub-Saharan Africa.
7	July 2024	Africa	Associate professor at a university and emergency care practitioner with previous research experience in AI for prehospital emergency care.
8	September 2024	Asia	Assistant professor in emergency medicine and hospital director of EMS with a special interest in data collection and AI solutions.
9	October 2024	Asia	Director of the capital city’s emergency medical centre and professor researching smart and AI emergency response systems.

Continents, instead of countries, are used to describe where the expert is based or has experience working to ensure anonymity for each expert.

Thematic analysis of the literature and expert consultations identified five overarching themes that need to be considered in the current utilisation and future development of AI in the PECS of LMICs. These themes are (1) the development of AI; (2) resource requirements; (3) data considerations; (4) human factors; and (5) local context. Table 2 provides an overview of the five themes identified and discussed in each included dataset. The findings related to each theme are discussed below and supported by tables displaying relevant quotes, which have been derived from included studies and the transcripts of expert interviews. Words in square brackets ([]) were not spoken by the expert/ written by the study author but have been included to indicate the topic of discussion and provide context.

**Table 2:** Themes identified and discussed in each included dataset.

Included datasets	Included themes				
	1: Development of AI	2: Resource requirements	3: Data considerations	4: Human factors	5: Local context
Studies					
Yang et al. (43)	✓	✓	✓	✓	X
Wang et al. (39)	✓	X	✓	X	X
Mapuwei et al. (46)	✓	X	X	✓	✓
Chen et al. (35)	✓	X	✓	X	X
Costa et al. (47)	✓	✓	✓	✓	✓
Rathore et al. (45)	✓	✓	X	X	X
Torres et al. (48)	✓	✓	X	✓	X
Yang et al. (42)	✓	X	✓	X	X
Z. Zhang et al. (41)	✓	X	✓	X	X
He et al. (36)	✓	X	✓	X	X
Ji et al. (38); 2019	✓	✓	X	✓	X
Boutilier and Chan (44)	✓	✓	✓	X	X
Huang et al. (37)	✓	✓	X	X	X
X. Zhang et al. (40)	✓	✓	X	X	X
Butsingkorn et al. (49)	X	✓	✓	X	X
Anthony et al. (50)	X	✓	✓	X	✓
Expert interviews					
ID 1	✓	✓	X	✓	X
ID 2	✓	✓	✓	✓	✓
ID 3	✓	✓	✓	✓	X
ID 4	✓	X	X	✓	✓
ID 5	✓	✓	✓	✓	✓
ID 6	✓	✓	✓	X	✓
ID 7	✓	✓	✓	✓	✓
ID 8	✓	✓	✓	✓	X
ID 9	✓	X	✓	✓	✓

### 3.1 Theme 1: development of AI

This theme, development of AI, explores how AI and its uses in prehospital care have developed over time and considers the directions they may continue to grow in. Some articles noted how AI technology has progressed to become more advanced and manage larger quantities of data (35, 39, 43, 46). Most authors discussed how the results of their studies can drive future AI applications and research (36, 38, 41–43, 45–48), with some studies forecasting the potential of combining AI tools with statistical or other AI models (37, 38, 40, 43, 44). Key quotes from these studies are shown in Table 3.

**Table 3:** Theme 1: development of AI quotes from literature review.

Theme 1: literature review quotes
*Combining deep learning and reinforcement learning, deep reinforcement learning is a revolutionary tool for artificial intelligence* (38). *There has been a shift of focus to the growing use and application of artificial neural network* (46). *Recent research has increasingly embraced advanced technologies such as deep learning, neural networks, and big data for forecasting patient volumes* (43). *For these models to be used in daily routine, some work still needs to be done, nevertheless, this work opens new lines of research* (42). *Due to the advances in computational capabilities, ANN [Artificial Neural Network] has been widely optimised and used in medical informatics in recent years* (35).

Experts also noted the progression towards more advanced AI technology (ID 1, ID 5, ID 7), although some acknowledged there is still a need for further refinement (ID 1, ID 7, ID 9). The majority of experts believed that AI has the potential to bring significant benefits to the PECS of LMICs (ID 1, ID 3, ID 4, ID 5, ID 6, ID 7, ID 8), and almost all experts interviewed discussed the various roles AI may play in this (ID 1, ID 2, ID 3, ID 5, ID 6, ID 7, ID 8, ID 9). Table 4 highlights some relevant quotes from the interviews. Consultations with experts also revealed a divide in opinion on the future progression of AI models in the context of LMICs. Some experts believed that explainability will be a key consideration in the implementation of AI in this context, including simpler models and the development of explainable deep learning AI models (XAI; ID 3, ID 4, ID 5, ID 8), while another expert believed that generative AI applications will transform future AI use in prehospital care (ID 1).

**Table 4:** Theme 1: development of AI quotes from expert consultation.

Theme 1: expert consultation quotes
*AI is something that very quickly went from something people were very excited about using – the possibilities are vast – to being afraid of it. (ID 3)* *I’m 100% optimistic about this technology but I do not think it’s the cure or the solution to every single problem. (ID 1)* *Every three to six months you get a model that is way better than the previous one. (ID 1)* *I think that the immediate gains, especially in low- and middle-income countries are going to be AI applications on efficiency and operations. (ID 5)* *There is a huge opportunity in low- and middle-income countries to actually get access to healthcare using this technology. (ID 1)* *As a caller is trying to explain something they switch over to another language for one or two words or one phrase. And I do not think AI is as agile to do that. (ID 7)* *As more users become available, the cost of AI will decrease and the barriers to access will be lower and lower. (ID 9)*

Overall, AI development is on a continued upward trajectory as the technology becomes increasingly complex and sophisticated. While there are some differences in opinion amongst experts around key considerations on the future direction of AI in prehospital care, subsequent research can leverage off previous studies to promote further innovation.

### 3.2 Theme 2: resource requirements

Some articles discussed how implementing AI solutions in EMS can reduce response times, save money and minimise resource use (37, 43, 45, 49) allowing for more equitable resource allocation (43, 44). As shown in Table 5, authors referred to the current lack of resources in LMICs as an important barrier to adequate prehospital emergency care (44, 45, 48–50). However, few studies considered the ability of countries to access the computational resources that would need to be involved to implement AI in some settings (38, 40, 45, 47). Moreover, Yang et al. (43) proposed using a statistical model in favour of AI to combat these computational demands.

**Table 5:** Theme 2: resource requirements quotes from literature review.

Theme 2: literature review quotes
*Accurate forecasting of emergency accidents can save time and resources, and reduce property damage and personal casualties. Besides, it is also helpful to optimise the limited resource allocation* (37). *South Africa, like most other African nations, has a shortage of emergency vehicles and advanced life-support providers who are equipped with the skills and experience in advanced decision-making* (50). *The computational models developed within the scope of this study were cost-effective, utilising only open-source resources that are freely available, which augments their feasibility for deployment in resource-constrained environments* (47). *However, as compared to rural regions, the rate of improvement in urban areas is substantially higher. This might be owing to the scarcity of both public and private ambulatory vehicles. Furthermore, the number of resources available to outsource non-ambulatory vehicles is restricted in rural areas* (45). *Estimating the expected travel time of an ambulance is crucial for the optimization of EMS. This is a non-trivial task, particularly in cities where funding is low and service provides must rely on their experience or consumer-level or open source mapping systems* (48).

Table 6 highlights some relevant quotes related to this theme from the expert interviews. Just under half of experts interviewed acknowledged the advantages of using AI to tackle resource limitations (ID 1, ID 2, ID 3, ID 5). Despite several experts noting that current IT infrastructure and expertise may be a limitation for using AI in some settings (ID 1, ID 2, ID 5, ID 8), these potential physical infrastructure challenges could potentially be overcome by leveraging off cloud architecture and mobile health solutions (ID 2, ID 5). Experts also considered the infrastructure required for adequate data security to protect detailed EHR as a potential limitation in the implementation of AI in LMICs (ID 3, ID 5, ID 7). In addition, experts noted the importance of funding and support for a project (ID 3, ID 5, ID 6, ID 8), with one expert admitting that the economy comes first (ID 3).

**Table 6:** Theme 2: resource requirements quotes from expert consultation.

Theme 2: expert consultation quotes
*We had a lot of technical difficulties getting this to work as these computers sometimes went down and we lost a lot of data, by having this parallel infrastructure instead of integrating our project into the architecture.* (ID 4) *That’s a lot of money saved for society as a whole. But that does not necessarily translate directly into the budget that you can see, so you have a great educational task of trying to explain why this prehospital initiative is actually going to save money down the line.* (ID 3) *How much money does it take to build it and test it versus how much revenue could I make?* (ID 6) *For low- and middle-income countries it could be a bigger problem, because in these cases you might not have the infrastructure for data security.* (ID 3) *You need somebody very skilled to do the statistics of the predictive AI piece. Not everyone can do that.* (ID 2) *You get a lot of efficiency gains and reduce the number of man-hours.* *Saves money, saves time and improves the quality of life for the staff.* (ID 5) *We should leverage off the mobile informatics revolution, where increasingly everybody has access to a smartphone.* (ID 5) *If you do not have funding for the project, it’s just shut down. I’m not sure how it is in your country, but a lot of projects end like this here.* (ID 8)

AI is generally considered to be able to optimise resource allocation in prehospital care. This is particularly important in LMICs that often lack appropriate levels of resources. However, other factors such as funding and infrastructure must be evaluated before attempting to implement any AI tools.

### 3.3 Theme 3: data considerations

Several studies noted that model precision could be improved with access to additional high-quality data (36, 42–44, 49, 50). With the majority of studies using retrospective datasets, some papers acknowledged the potential for bias in study design and participant selection (35, 39, 41, 42, 47). Minimising inherent biases in data collection methods and ensuring models are only trained on high quality data that represents the population it is serving, were seen by some as two key processes to limit the effect of data bias (39, 43, 47, 50). Relevant quotes from the literature are included in Table 7.

**Table 7:** Theme 3: data considerations quotes from literature review.

Theme 3: literature review quotes
*Additional data could significantly enhance the development of a prediction model, ensuring both greater precision and reliability* (49). *The retrospective and observational design inherits potential for bias. Our results need to be confirmed prospectively in the prehospital emergency cohorts* (39). *There is a selection bias in our study population because some stroke patients who were not recognised by paramedics during the pre-hospital stage were not included in the scope of our observation* (41). *The sensitivity and specificity of the RFs [Random Forest] model for LVO might differ from pre-hospital cohorts with suspected stroke that include stroke mimics and haemorrhagic strokes. Thus, we cannot rule out a selection bias* (39). *Artefacts produced by chest compression may preclude reliable rhythm analysis and accurate VF waveform analysis during CPR, and therefore degrade the prediction accuracy* (36).

Expert opinions were largely congruent with study authors on the importance of, and how to limit data bias (ID 2, ID 3, ID 5, ID 7, ID 8, ID 9). Access to a high quality and quantity of representative data was considered amongst experts to be one of the most significant limitations to the implementation of AI in PECS in LMICs (ID 2, ID 3, ID 5, ID 7, ID 9). Some experts commented on the need for LMICs to digitise health records before they can embrace AI solutions (ID 5, ID 7, ID 8). Most experts warned of the risk of data bias and the consequences this could have on specific populations (ID 2, ID 3, ID 5, ID 6, ID 7). Examples of applicable quotes can be found in Table 8.

**Table 8:** Theme 3: data considerations quotes from expert consultation.

Theme 3: expert consultation quotes
*You need a completeness in representation – a representative dataset I would say, is actually more important than completion.* (ID 3) *You can have the most powerful large language model or processing power in the world. But if you are punching faulty data or the data is already inherently bias, it’s not going to help you very much.* *In fact, it may lead you down the wrong path.* (ID 5) *Data quality is always the biggest limitation for anything AI. Data quality is key and when I say quality – its quantity and quality.* (ID 2) *Say for example, we only use the data set of high income, high education men in their forties and above. Then you have a biased dataset which only helps the population in this dataset.* (ID 3) *Garbage in, garbage out. So there is lots of data around, but the problem is a lot of it is noise.* (ID 5) *We still have a problem with how we collect data because in most areas, we still use paper and not computers.* (ID 8) *The core problem is validation in the field of prehospital emergency care.* (ID 9)

AI model accuracy can be improved by increased focus on data collection procedures. This includes ensuring you collect a large quantity of quality data, and that it is debiased, so that it better represents the population. LMICs aiming to use AI in their prehospital system, can facilitate algorithm access to larger quantities of data by digitising their existing health records.

### 3.4 Theme 4: human factors

Some studies advise that increased collaboration between researchers, developers and healthcare workers will increase the chance of successful implementation (43, 46, 47). However, factors such as human error in records, can also limit the effectiveness of AI (48). Table 9 highlights some applicable quotes around this subject and wider theme. One study accommodated for some human factors, such as ignorance towards AI decisions, to more closely resemble the differences in experiments and real life (38).

**Table 9:** Theme 4: human factors quotes from literature review.

Theme 4: literature review quotes
*Rather than replacing human judgement, our proposal seeks to complement it, potentially enhancing healthcare professionals’ efficiency and accuracy in critical situations* (47). *We investigate the situations in which crews in ambulances disobey or delay the redeployment decisions obtained by our method* (38). *The reasons behind a prediction need to be understandable by physicians and patients; thus, for ML models in the medical field that interpretability is a core requirement* (42). *These records are highly susceptible to human error and omission, particularly since they are filled by the paramedics at the scene, which, given the lack of personal and equipment, have an overburdened workload that makes bookkeeping a low priority task* (48).

A majority of experts believe all AI tools should be designed with the human end-user in mind, as model precision can be negatively impacted by human decisions in real life (ID 2, ID 3, ID 4, ID 5, ID 7, ID 9). As demonstrated in some of the quotes shown in Table 10, almost all experts stated that they believe AI tools should be developed to work alongside humans and not replace them (ID 1, ID 2, ID 3, ID 4, ID 5, ID 7, ID 9). To ensure the successful implementation of AI tools into a healthcare system, most experts feel that there is a need to educate the end-user on the purpose and functioning of the model (ID 3, ID 4, ID 5, ID 7). This was believed to build trust in the technology amongst the users (ID 2, ID 3, ID 4). Some experts maintained that XAI can also establish trust in AI (ID 3, ID 5, ID 8). Enhancing collaboration between contributors on future projects was also seen by some experts as an important step towards improving AI tools (ID 4, ID 5, ID 7, ID 8).

**Table 10:** Theme 4: human factors quotes from expert consultation.

Theme 4: expert consultation quotes
*Even though the model was perfect in the prospective trial and the randomised trial, we did not have the effect we aimed for because the dispatchers did not comply with the AI suggestions that were given.* (ID 4) *My colleagues feel they need to understand more about the mechanisms that machine learning uses to generate results, so they are afraid to use the results on the patient.* (ID 8) *This is not replacing a person; this is helping a person, so you can do more and you can do it quicker.* (ID 2) *I think when we are picking which area we should work on, we should look at trying to find the areas where people actually want some help.* (ID 4) *The person that uses the AI must have the requisite training to understand the [AI’s] shortcomings, when they should override it, when they should trust it and how they should trust it.* (ID 7) *You cannot expect a four-week trained ambulance driver to be able to give the depth and quality of data that you might need to do mortality predictions.* (ID 7) *Who has the final decision or power and who is responsible when things go wrong?* (ID 3) *We have differences in the community between people who have digital literacy and people who do not. I think these people [without digital literacy] are concerned about this.* (ID 8)

Overall, integrating humans in the decision process of AI models is necessary to ensure successful implementation, but this can limit the accuracy of the model. To minimise the risk of this occurring, all AI models should be developed using human-centred design principles.

### 3.5 Theme 5: local context

Table 11 displays relevant quotes related to this theme from the literature review. To ensure AI models benefit the population they have been built to help, they all should incorporate local context into their design and function. However, this is particularly important for models that deal with spoken language (47, 50). Some studies also discussed the importance of using contextually appropriate model designs (46, 47, 50).

**Table 11:** Theme 5: local context quotes from literature review.

Theme 5: literature review quotes
*This study can be applied to different parts of the country with modifications due* var*iation in traffic conditions and norms of EMS* (45). *South Africa, like many other African nations, has multiple spoken languages. These languages, sometimes, differ substantially in their grammatical structure and syntax* (50). *It is not the norm, and often not possible due to congestion, for motorists to yield for emergency vehicles* (44). *This migration trend has seen the increase of unemployment in urban areas, sprouting of new residential areas, both formal and informal* (46). *Unlike English, Chinese words are constructed differently, requiring the use of a word splitting tool to segment the entire sentence* (40).

Experts were keen to stress models should only be implemented for the local population they have been trained on, including considerations on important differences in urban and rural populations (ID 5, ID 6, ID 7). Applying the model outside of the local context would require retraining (ID 2, ID 4). Some experts believe social and cultural acceptability must be considered when implementing AI tools (ID 5, ID 6, ID 7, ID 9). Key quotations related to this theme are included in Table 12.

**Table 12:** Theme 5: local context quotes from expert consultation.

Theme 5: expert consultation quotes
*[Monoculture is] the export of high-income country culture mostly through media. So AI is trained on monoculture and then you are trying to apply it to multicultural contexts. For it to have utility in our context it actually needs to be multicultural and not monocultural.* (ID 7) *We built the system here; we were not importing a system or anything like that. I think obviously that’s so important.* (ID 6) *If the AI is very language specific or very population specific you cannot use it everywhere, but the same model can be used. You just need to go through the data and training process.* (ID 2) *We cannot make recommendations for LMICs, it’s impossible. You cannot because it’s too diverse. The angle of LMICs and the level of development, within the emergency care system in particular, is completely different.* (ID 7) *You need to retrain these things every now and then. And everybody needs to know this when you have implemented it, it’s not just once. You have to keep readjusting your models to the changing reality.* (ID 4) *The AI might be accurate, but it might make inappropriate or irresponsible recommendations. And that’s why it has to understand the full context of what you are doing.* (ID 7) *Adaptations to artificial intelligence have to be based on local economic, social and cultural traditions.* (ID 9)

In conclusion, factors such as language and culture cannot be ignored when designing an AI tool. These and other context-specific factors must be taken into consideration to ensure successful implementation.

## 4 Discussion

In this study, 16 studies and the transcripts of nine interviews with field experts were thematically analysed to assess the current state of AI utilisation in PECS in LMICs and the implications for development. The findings of this study suggest AI can be an effective tool to use in these settings, however the key areas that researchers and clinicians implementing AI tools should consider are the rapid and constant development of this technology; the importance of high quality, unbiased data; any gaps in current and required resources for implementation; the incorporation of human-centred design into the project; and the importance of ensuring models are acceptable within the local context. This discussion will review the findings from this study and discuss the success factors and challenges in implementing AI models in the PECS of LMICs. By building on the results of this study, this discussion will seek to outline important considerations for a future strategic vision in this field.

### 4.1 Ensuring high-quality data

AI tools in PECS should have the overarching goal to improve patient outcomes by facilitating the treatment of patients as soon as possible after an acute injury or illness occurs. The results of this qualitative analysis suggest that attaining adequate amounts of high-quality data is fundamental to achieving this goal. However, acquiring the necessary level of detail in data can be difficult for some LMICs (51). Healthcare systems in some of these countries do not have access to personnel with adequate training in data collection and analysis (52). This is typically exacerbated in PECS, many of which often lack trained medical personnel (14). Ensuring workers are skilled and available to appropriately use AI tools has been a challenge in adopting AI previously (53). Even when personnel are appropriately trained, they can lack the system-wide technical infrastructure and therefore have limited capacity to use data on the frontline (52, 54). Organisational challenges such as financial limitations and inadequate government policies further limit accurate data collection (52, 55). In addition to facilitating the integration of AI, addressing structural barriers is important as LMICs seeking to improve the delivery of healthcare generally may benefit from improvements in data utilisation through a cyclical positive feedback process (54). By focusing on improving data utilisation as a means to improve a healthcare system, healthcare services seek to gather additional data. This demand can promote improvements in data collection and analysis methods, through technical infrastructure, training of individuals to ensure correct procedural technique, and additional organisational support (52, 56). The digitisation of written records into EHR is one way to support improved data collection and analysis (52). This should be a priority for health systems in LMICs to allow AI technology to be appropriately trained (57). Electronic healthcare systems can also lower costs, boost transparency and lead to efficiency gains (54, 58). These adaptations can result in an increase in information available for stakeholders to make informed decisions. Improving access to this information can allow more stakeholders to use all the available information to make more evidence-based health decisions, thereby promoting evidence-based policies (56). Adopting policies supported by research is seen as essential for the development of emergency care in LMICs (59). Finally, a greater desire for information to assist decision-making stimulates a greater demand for data, allowing the cycle to continue (56).

AI can also widen the scope of information available for evidence-based decisions by improving the efficiency and accuracy of data analysis processes by rapidly identifying patterns from large volumes of data (22). However, it is important that the use of information, and therefore the demand for data (56), is based on the needs of the population and not on the current availability of data and information (5). This can be accomplished by implementing AI models that are designed to address the specific and relevant challenges in prehospital care for a defined population or setting. Aside from the advantages directly associated with using AI in LMICs and the positive feedback mechanism discussed, upgrading existing digital healthcare infrastructure to meet the data demands of AI will likely result in other wider benefits for the health system. One example of this is cloud computing, which can provide leverage for AI and simplify its deployment in a healthcare system (60), while simultaneously improving the reliability and reducing the cost of data storage and management (1). Despite these benefits, many LMICs lack the sustained financial resources for AI implementation, with funding being prioritised for wider EMS upgrade costs instead (61). If executed correctly, AI augmentation may reduce healthcare costs through improvements in operational efficiency and quality care. Resource limited settings should prioritise areas that are lower cost, offer the greatest gains in efficiency and target diseases with the highest burden to maximise AI investment and simultaneously upgrade PECS (58). However, this can be expensive to undertake and countries implementing AI tools in PECS should consider both direct and indirect costs with this technology, ensuring rigorous procedures are in place to allow for sustained cost savings and efficiencies (62).

### 4.2 Human-centred approach

According to the study results, continued technological innovation is believed to improve AI models in PECS in LMICs. The ability to use XAI without limitations to performance is considered by some to play a central role in future applications by making currently hidden deep learning decision processes visible, promoting model reliability and physician trust (57, 63). Future AI models should not only demonstrate transparency in how the data is used to generate a final decision but should also clearly show where the data has been obtained. Algorithms based on data from a HIC will likely not reflect the population of a LMIC, and risk exacerbating established prejudices (64, 65). Even if data is collected from the population of a selected LMIC, factors such as aggregation bias or representation bias can ensure the collected data is not a true reflection of the population. Moreover, making an assumption about a single patient based on observations of an entire population is unlikely to properly accommodate for individual differences (66, 67). For example, if an AI tool to predict sepsis is based on data from the general population, a patient with a compromised immune system may be screened as healthy by the algorithm but be, in reality, very unwell. The model trained using observations from “typical” patients, i.e., immunocompetent patients, is unlikely to recognise an immunocompromised patient as unwell. Within local populations, vulnerable and marginalised groups often face more significant challenges in accessing technologies such as AI, meaning populations with a perceived higher social standing are more likely to be able to access and therefore benefit from new health innovations (68). Not only does this result in improved outcomes only for these patients, but the resulting algorithm is also trained and refined based on this sample, and therefore introduces bias into the tool and becomes more accurate for the convenient majority, potentially negatively impacting patient outcomes for other historically marginalised demographic groups (66, 69). To minimise this risk, AI tools and the corresponding algorithms must be regularly audited by independent third parties. These auditing processes should be continuous and adapted to appropriately assess the AI tool as it is developed and refined. It is essential that these evaluations assess the model’s performance within these vulnerable communities (70). These processes should follow the framework elements and principles outlined in the World Health Organisation’s guidelines on AI ethics and governance (70).

When correctly integrated into a specific setting, AI can improve the quality of and access to healthcare for some populations (65). For example, using AI may be particularly useful in multicultural settings where patients speak a diverse set of languages by providing real-time accurate translations of discussions between healthcare provider and patient. Some healthcare professionals view AI as potentially beneficial for patients who are normally isolated by language barriers, by improving the standard of care for these patients and providing additional support to the healthcare team, on top of gains in efficiency (71). This function can have further unintended benefits for health systems and staff as well. For dispatch services, AI may also reduce levels of staffing required to address language barriers, relieving emergency care teams in resource-limited settings (72). Moreover, automating certain processes in emergency care can even lead to improvements in job satisfaction amongst staff, by lowering levels of frustration and the perceived time spent documenting notes (73).

### 4.3 Ethical considerations

Although an essential factor in AI development, ethical considerations for equitable AI use should not be limited to ensuring representative datasets, but should also consider the availability of equipment and staff in the healthcare system (69). For example, should an AI tool advise intubation for a prehospital patient if there are no healthcare personnel available with the skills and knowledge to intubate? What if there are no intensive care beds available for the patient when they arrive at hospital? There is little advantage in improving the ability to diagnose a condition if a particular healthcare system lacks the resources to treat it (1). Ethical implications of AI use in the prehospital setting should be discussed and decisions made with consideration for current system capacity, as well as local culture and customs (69). This point has been previously disregarded in the technological research agendas implemented by Western actors that lack local contextual knowledge, who instead seek to engage and satisfy the needs of international donors more than those of the end-users (74).

Healthcare systems in LMICs that are seeking to implement AI will also need to address data security and patient privacy concerns that accompany the generation of electronically stored datasets (1, 13). AI models are reliant on multiple actors accessing patient information across different databases (13), potentially exposing confidential medical records to malicious threats (52). Previous studies have highlighted the difficulties of establishing privacy policies and enforcing medical law in LMICs (75). Although these issues exist in AI use in all areas of medicine, the diversity of patient presentations in PECS makes it difficult to develop generalised regulations for this domain. In addition, the imminently life-threatening nature of some patient presentations means that the ease of access to vital patient information such as past medical history and drug allergies is an important consideration when developing security measures for EHR. Despite this, the ease of accessing data must never be at the expense of patient confidentiality. AI developers will have to balance these ethical privacy challenges with the accessibility requirements of healthcare staff (1). Governments of LMICs will also have to manage energy security concerns if AI is implemented in the healthcare system. AI systems are reliant on large amounts of energy to process information through its servers, that then require cooling with clean, fresh water. Although some studies suggest AI can improve energy efficiency in LMICs (76), this computational demand combined with the rapid growth of large language models is expected to exceed any potential energy savings in the coming years (77). Balancing this net-positive energy expenditure of AI, coupled with growing international concerns about the effect of greenhouse gas emissions on climate change, against the possible advantages of implementing AI tools, will be a challenge for LMICs as they continue to rely heavily on fossil fuels and struggle to match energy supply with demand (76). Power cuts affecting AI tools that PECS have become reliant on may have devastating consequences for patient outcomes.

### 4.4 Future directions

There is a need to successfully implement AI now so that it can continue to benefit society in the future. This is particularly important in LMICs, where preliminary research is demonstrating AI’s potential to augment current health service distribution (5). To address some of the current issues with AI utilisation in PECS in LMICs, there must be improved international partnership between stakeholders in prehospital EMS, government organisations, research and the medical technology field (15), to empower leadership from those with local, contextual experience to implement necessary changes. This will promote the safe, ethical and socially acceptable use of AI technology and ensure a smooth transition towards programs funded and run by local people (23). Collaboration should occur early in the design process, allowing governments and investors to make informed decisions about the implementation of this technology and strengthen the sustainability of the intervention (21). These discussions should continue after the implementation of the device, to make sure algorithms are appropriately retrained and adapt to the changing reality of their environment (51). All collaborators should ensure understanding in a tool’s applications and limitations, and should share collective responsibility for any implemented AI models (70). By ensuring humans remain central to decisions made while using AI, healthcare systems can also ease legal liability and ethical considerations in cases of patient harm (1, 57).

Stakeholders involved in these discussions should also develop a regulatory framework for AI use in these settings. This should set out the legal requirements for this technology to prevent models discriminating against marginalised populations (69). This framework should also consider how to ensure data is securely stored (52). Having governmental support for AI-friendly policies can further motivate healthcare systems to embrace AI solutions (78). In addition, prehospital emergency care staff that will avail of this technology should be educated on how any future AI tools will work before they have been implemented and used. Improvements in training should also be extended to those involved in data collection and analysis, with an emphasis on transparency at all stages of data collection, model training and implementation to make sure standards are upheld, evidence-based decision making processes are followed, and future developers learn from previous mistakes (12, 69, 79). There is also a need for more qualitative reviews of key actors, including patients, on the use of AI in prehospital settings. This should include studies on the acceptance of AI amongst key prehospital staff in LMICs, as well as how to ensure contextual acceptability.

### 4.5 Limitations

This study had several limitations. Firstly, participants may have felt obligated to respond to questions in a socially acceptable way instead of sharing their true opinions. To try and limit this, the interviewer used a semi-structured approach with open and follow-up questions. Secondly, as participants were unable to provide feedback on the findings and therefore validate the results, it must be acknowledged that the results are based on the interviewer’s interpretation of the expert’s spoken word and may not directly mirror the intention of the expert. Using a single coder to identify themes in the data can also introduce the risk of interpretive bias in the analysis. To try and reduce this risk of bias, themes were discussed with all co-authors to ensure inter-author agreement. Finally, the sampling methods of snowball and purposive sampling mean the study sample may not be representative of the range of researchers and clinicians involved in this research area, reducing the external validity of the findings. Moreover, only nine participants were interviewed for the thematic analysis, possibly limiting the depth of identified themes. However, as this research area is only beginning to develop there are only a limited number of experts working on AI in PECS in LMICs, some of whom may not have fully developed strong international research links, making it difficult to identify experts through the selected sampling methods. This is also reflected in the geographical representation of interviewed experts. Most participants originate from Europe and Asia, with other LMIC regions such as Latin America underrepresented. This sampling bias may limit the applicability of the study’s findings in these underrepresented areas. This lack of expert availability is one of the reasons a scoping review method was applied, as it would be difficult to achieve true data saturation.

## 5 Conclusion

AI is seen by some to be a key missing piece in the drive towards universal health coverage and the realisation of the United Nations’ Sustainable Development Goal 3: Good Health and Wellbeing (13, 79, 80). Improvements across all areas of medicine, but particularly in emergency care will be an essential step to achieve this ambitious target (81). LMICs and low-resource settings may be in a good position to improve their public health systems by sustainably implementing this technology (1). Moreover, AI has the potential to facilitate the improvement of data collection and management procedures, enhancing overall data quality and ensuring healthcare decisions are evidence-based. However, AI will not provide the solution many people hope without consideration for its future directions in development; resource requirements; data needs; human factors; and local context. Without appropriate initial infrastructure to support its use, AI technology cannot be a long-term solution in prehospital settings. Stakeholders must also consider the ethical implications of introducing this technology into the healthcare system. These targets can be achieved through improved collaboration between all involved stakeholders, led by those with context-specific knowledge and experience. This partnership can promote creation of a regulatory framework to ensure AI is used responsibly, as well as provide further education for the healthcare staff using this technology. Continued research into AI use in PECS, particularly within LMICs, involving both qualitative and large, prospective and randomised quantitative studies is required. As the field of prehospital AI continues to develop and the perceived benefits become more urgently needed, it is essential that future developers, researchers and EMS staff reflect on the success factors and challenges of previously implemented AI tools to ensure that future applications are sustainable for the future and benefit the patient, staff and wider healthcare system.

## Data Availability

The raw data supporting the conclusions of this article will be made available by the authors, without undue reservation.
