# Supplementary material for: Artificial intelligence in prehospital emergency care systems in low- and middle-income countries: cure or curiosity? Insights from a qualitative study
Source: Front Public Health. 2025 Oct 1;13:1632029. doi: 10.3389/fpubh.2025.1632029 (PMC12521113; doi:10.3389/fpubh.2025.1632029)
Supplement: Supplementary file 1 [file Supplementary_file_1.docx]

1. **Supplementary Figure and Tables**
   1. **COREQ Checklist**

**COREQ (COnsolidated criteria for REporting Qualitative research) Checklist**

A checklist of items that should be included in reports of qualitative research. You must report the page number in your manuscript where you consider each of the items listed in this checklist. If you have not included this information, either revise your manuscript accordingly before submitting or note N/A.

| **Topic** | **Item No.** | **Guide Questions/Description** | **Reported in Section** |
| --- | --- | --- | --- |
| **Domain 1: Research team and reflexivity** |  |  |  |
| *Personal characteristics* |  |  |  |
| Interviewer/facilitator | 1 | Which author/s conducted the interview or focus group? | Methods |
| Credentials | 2 | What were the researcher’s credentials? E.g. PhD, MD | Methods |
| Occupation | 3 | What was their occupation at the time of the study? | Methods |
| Gender | 4 | Was the researcher male or female? | Methods |
| Experience and training | 5 | What experience or training did the researcher have? | Methods |
| *Relationship with participants* |  |  |  |
| Relationship established | 6 | Was a relationship established prior to study commencement? | Methods |
| Participant knowledge of the interviewer | 7 | What did the participants know about the researcher? e.g. personal goals, reasons for doing the research | Consent for Publication |
| Interviewer characteristics | 8 | What characteristics were reported about the interviewer/facilitator? e.g. Bias, assumptions, reasons and interests in the research topic | Methods |
| **Domain 2: Study design** |  |  |  |
| *Theoretical framework* |  |  |  |
| Methodological orientation and Theory | 9 | What methodological orientation was stated to underpin the study? e.g.  grounded theory, discourse analysis, ethnography, phenomenology, content analysis | Methods |
| *Participant selection* |  |  |  |
| Sampling | 10 | How were participants selected? e.g. purposive, convenience, consecutive, snowball | Methods |
| Method of approach | 11 | How were participants approached? e.g. face-to-face, telephone, mail, email | Methods |
| Sample size | 12 | How many participants were in the study? | Methods, Table 1 |
| Non-participation | 13 | How many people refused to participate or dropped out? Reasons? | Methods |
| *Setting* |  |  |  |
| Setting of data collection | 14 | Where was the data collected? e.g. home, clinic, workplace | Methods |
| Presence of nonparticipants | 15 | Was anyone else present besides the participants and researchers? | Methods |
| Description of sample | 16 | What are the important characteristics of the sample? e.g. demographic data, date | Table 1 |
| **Topic** | **Item No.** | **Guide Questions/Description** | **Reported on Page No.** |
| *Data collection* |  |  |  |
| Interview guide | 17 | Were questions, prompts, guides provided by the authors? Was it pilot tested? | Methods, Supplementary Material |
| Repeat interviews | 18 | Were repeat inter views carried out? If yes, how many? | Methods |
| Audio/visual recording | 19 | Did the research use audio or visual recording to collect the data? | Methods |
| Field notes | 20 | Were field notes made during and/or after the inter view or focus group? | Methods |
| Duration | 21 | What was the duration of the inter views or focus group? | Methods |
| Data saturation | 22 | Was data saturation discussed? | Methods |
| Transcripts returned | 23 | Were transcripts returned to participants for comment and/or correction? | Methods |
| **Domain 3: analysis and findings** |  |  |  |
| *Data analysis* |  |  |  |
| Number of data coders | 24 | How many data coders coded the data? | Methods |
| Description of the coding tree | 25 | Did authors provide a description of the coding tree? | N/A |
| Derivation of themes | 26 | Were themes identified in advance or derived from the data? | Methods |
| Software | 27 | What software, if applicable, was used to manage the data? | Methods |
| Participant checking | 28 | Did participants provide feedback on the findings? | Methods |
| *Reporting* |  |  |  |
| Quotations presented | 29 | Were participant quotations presented to illustrate the themes/findings?  Was each quotation identified? e.g. participant number | Results |
| Data and findings consistent | 30 | Was there consistency between the data presented and the findings? | Results |
| Clarity of major themes | 31 | Were major themes clearly presented in the findings? | Results, Table 2 |
| Clarity of minor themes | 32 | Is there a description of diverse cases or discussion of minor themes? | Results |

Developed from: Tong A, Sainsbury P, Craig J. Consolidated criteria for reporting qualitative research (COREQ): a 32-item checklist for interviews and focus groups. *International Journal for Quality in Health Care*. 2007. Volume 19, Number 6: pp. 349 – 357

**Once you have completed this checklist, please save a copy and upload it as part of your submission. DO NOT** **include this checklist as part of the main manuscript document. It must be uploaded as a separate file.**

**1.2** **Figure 1. PRISMA flow diagram for literature search.**

Diagram showing the Preferred Reporting Items for Systematic Reviews and Meta-Analysis – Extension for Scoping Reviews (PRISMA-ScR) flow diagram. Adapted by the study author based off PRISMA flow diagram.

*Template From:* Tricco AC, Lillie E, Zarin W, O'Brien KK, Colquhoun H, Levac D, et al. PRISMA Extension for Scoping Reviews (PRISMAScR): Checklist and Explanation. Ann Intern Med. 2018;169:467–473. [doi: 10.7326/M18-0850](http://annals.org/aim/fullarticle/2700389/prisma-extension-scoping-reviews-prisma-scr-checklist-explanation).

**
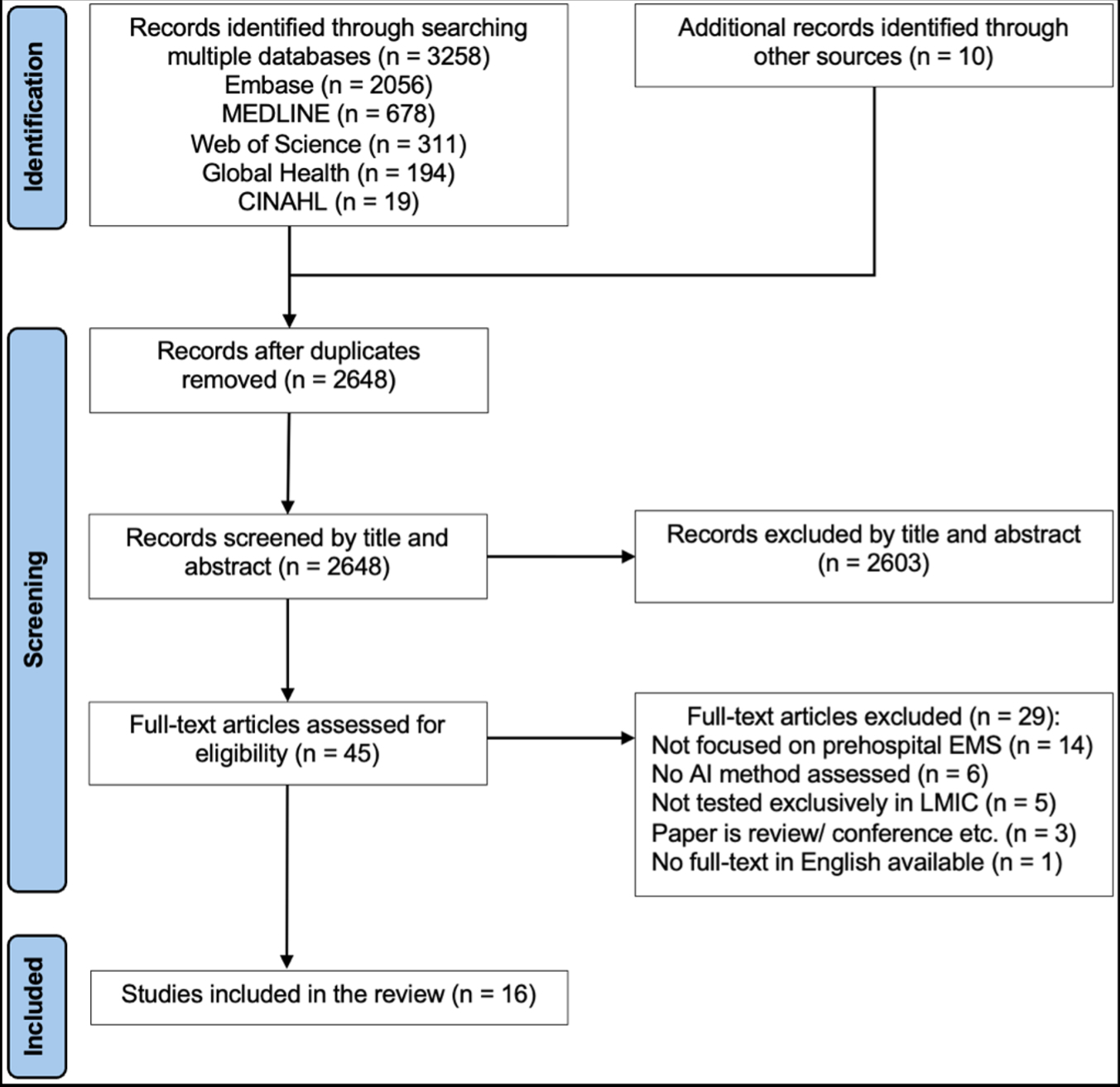
***Adapted Diagram From:* Mallon, O., Lippert, F., Stassen, W., Ong, M. E. H., Dolkart, C., Krafft, T., & Pilot, E. (2025). Utilising artificial intelligence in prehospital emergency care systems in low- and middle-income countries: a scoping review [Systematic Review]. *Frontiers in public health*, *Volume 13 - 2025*. <https://doi.org/10.3389/fpubh.2025.1604231>

- 1. **Semi-Structured Interview Guide**

Interview guide to be used by researcher in study Using Artificial Intelligence in Prehospital Emergency Medical Services in Low- and Middle-Income Countries: A Scoping Review

**Questions**

- Tell me about your background – your occupation, experience and training.
  - What is your previous experience working in prehospital emergency medicine?
  - What is your previous experience working with artificial intelligence?
  - What are some projects you are currently involved in this area?
- Describe the process for developing an AI model in healthcare. For example: the conception, the AI tool, the data collection, the training, the implementation.
- Does funding play a major role in ensuring AI tools are successfully implemented into health systems?
  - How do you ensure that there is adequate funding to support projects?
- How important is it to collect high quality data to train artificial intelligence models? Why is it important?
- What are some problems that occur during data collection that can affect the quality of the data?
- How do you minimise/ reduce the risk of bias in collected data?
- How do you ensure that an AI tool can be successfully implemented into a prehospital healthcare system?
- What are the most important aspects to consider when creating a successful AI model for the prehospital emergency healthcare services of LMICs?
- If I wanted to implement an AI tool in a prehospital system in an LMIC (e.g., China), what are some of the key pitfalls or challenges that I should be aware of?
- To what extent should we consider the human end-users of this technology in its design?
- How do you minimise the risk of harm to patients and staff from AI?
- Should AI models be adapted to the local context/ population?
  - If so, how should they be adapted?
- Does the sociocultural context play a role in implementing AI?
- What are some ethical concerns surrounding AI use in this area?
- How do you think AI models in emergency care will continue to develop? What is the future of AI in this field?
  - Will interpretable models (explainable AI) play a big part in the future? Why?
  - What will AI be used for in the future?
- What are the gaps in current research into this topic?
  - Is there a need for more retrospective/ prospective/ randomised studies?
  - What areas of prehospital care can AI address?
- Are there any remaining topics or issues you would like to comment on?
